# A prognostic nomogram for predicting overall survival in gastric signet ring cell carcinoma patients: a SEER database and Chinese registry analysis

**DOI:** 10.3389/fmolb.2025.1704157

**Published:** 2025-11-17

**Authors:** Jie Wu, Jichen Wang, Ning Chen, Junjie Nie, Ling Xia, Quanpeng Li, Xueting Deng, Guozhong Ji

**Affiliations:** Second Affiliated Hospital of Nanjing Medical University, Nanjing, China

**Keywords:** gastric signet ring cell carcinoma, gastric cancer, SEER database, nomogram, survival analysis, lymph node ratio

## Abstract

**Purpose:**

Gastric signet ring cell carcinoma (GSRC) is a distinct gastric cancer (GC) subtype. This study aimed to develop and validate a nomogram to predict overall survival (OS) and guide clinical decision-making.

**Methods:**

This study included 2,203 GSRC patients from the SEER database (2010–2019), randomly split into a modeling cohort (n = 1,542) and an internal validation cohort (n = 661). An external cohort of 74 patients from the Second Affiliated Hospital of Nanjing Medical University (2019–2024; median follow-up 34 months) was used for validation. Predictor variables—age, sex, chemotherapy, lymph node ratio (LNR), T and M categories, tumor size, and tumor number—were included in a cox proportional hazard model. A nomogram was derived from the cox model and internally validated using 1,000 bootstrap resamples. Discrimination, calibration, and decision curve analysis (DCA) evaluated model performance.

**Results:**

The nomogram included age, chemotherapy, LNR, T and M categories, and tumor size. In the modeling cohort, time-dependent area under the receiver operating characteristic curve (AUC) was 0.79, 0.85, and 0.85 at 12, 36, and 60 months; internal validation AUCs were 0.79, 0.85, and 0.85. In the external cohort, AUC at 36 months was 0.91 (primary horizon), with exploratory IPCW-AUCs of 1.00 at 12 and 60 months due to class imbalance. Calibration showed close agreement between predicted and observed OS, and DCA demonstrated clinical net benefit across relevant thresholds.

**Conclusion:**

This study developed a nomogram for OS prediction in GSRC patients, supporting risk stratification and clinical decision-making.

## Introduction

Gastric cancer (GC) is a major health challenge worldwide. According to the World Health Organization’s 2022 global cancer statistics, nearly 1 million new cases and around 700,000 deaths were reported annually, making it the fifth leading cancer in both incidence and mortality (Bray et al., 2024). Gastric adenocarcinoma is the most common type of GC, accounting for about 90% of all cases (Lopez Sala et al., 2023). Within the World Health Organization classification system, gastric signet ring cell carcinoma (GSRC) is recognized as a unique histological subtype of gastric adenocarcinoma, which accounts for about 17% of all GC and over half of all signet ring cell carcinoma (SRCC) (Tang et al., 2023). GSRC is primarily composed of signet ring cells, which contain a large amount of mucin that squeezes the nucleus to the cell periphery, giving the cell a distinctive ‘signet-ring’ appearance (Chen J. et al., 2023; Benesch and Mathieson, 2020).

GSRC is characterized by poor differentiation and is associated with a high risk of metastasis and aggressive behavior (Bray et al., 2024; Zhao et al., 2023). The difference in prognosis between GSRC and non-GSRC remains debatable. A large-scale study of over 10,000 cases of GSRC and non-GSRC cases found that GSRC was not an independent prognostic factor for advanced GC, but it was associated with more aggressive tumor behavior (Taghavi et al., 2012). Several studies have indicated that GSRC patients tend to have a relatively better prognosis in the early stages but a poorer prognosis in the advanced stages compared to those with other histological subtypes, largely because GSRC is often diagnosed at a more advanced stage (Zaafouri et al., 2022; Piessen et al., 2009). Factors like patient age, tumor size, tumor-node-metastasis (TNM) stage, epidermal growth factor receptor (EGFR) status, treatment choices can all impact the prognosis of GSRC (Li et al., 2022; Boot et al., 2025; Efared et al., 2020; Huang et al., 2024; Xie et al., 2022; Liu et al., 2023). Although the global incidence of GC has declined due to increased awareness of *Helicobacter pylori* eradication and improvements in early detection (Smyth et al., 2020; Sun et al., 2023), the incidence of GSRC has been steadily rising in Asia, the United States, and Europe, with some studies reporting that GSRC accounts for approximately 15.1%–45% of newly diagnosed GC cases (Graziosi et al., 2023; Nie et al., 2022; Li et al., 2019; Liu et al., 2024). These trends highlight the urgent need to investigate the prognostic models and associated prognostic factors of GSRC to improve diagnostic accuracy and individualized prognostic evaluation.

The Surveillance, Epidemiology, and End Results (SEER) database collects cancer incidence data from population-based cancer registries covering approximately 45.9 percent of the U.S. population. It provides a large-scale and multicenter data foundation for research on rare tumor subtypes, making it an essential platform for studying GSRC (Altekruse et al., 2014; Che et al., 2023).

Nomograms, which are simple and visual prediction tools, have been gaining popularity in cancer study. In this study, we used clinical data from the SEER database and the Second Affiliated Hospital of Nanjing Medical University to build a large and representative GSRC patient dataset. Our goal is to evaluate prognostic factors and further develop a reliable model to predict overall survival (OS) in GSRC patients. This model is expected to provide an effective tool for individualized clinical management and decision-making support in GSRC patients.

## Methods

### Modeling and internal validation cohorts

Clinical data were extracted from the SEER database using SEER*Stat software (version 8.4.5). Patients included from the SEER database met the following criteria: (1) histological confirmation of GSRC based on the International Classification of Diseases for Oncology, Third Edition (ICD-O-3) histology code 8,490; (2) primary tumor located within the stomach (ICD-O-3 site code C16.0-C16.4, C16.8); (3) diagnosed between 2010 and 2019; and (4) completion of radical gastrectomy. The exclusion criteria were: (1) presence of concurrent primary malignancies; and (2) missing or unknown clinicopathological information.

Following the application of the criteria, a total of 2,203 patients were included in the analysis. This complete-case approach was chosen because internal consistency across variables was prioritized. No multiple imputation was performed.

The 2,203 patients were randomly divided into a modeling cohort (70%, n = 1,542) and an internal validation cohort (30%, n = 661).

### External validation cohort

An external validation cohort was assembled retrospectively from GSRC patients who underwent radical gastrectomy at The Second Affiliated Hospital of Nanjing Medical University between January 2019 and December 2024. The inclusion criteria were: (1) postoperative pathological confirmation of GSRC; (2) primary tumor originating in the stomach; and (3) completion of radical gastrectomy. The exclusion criteria were: (1) presence of concurrent primary malignancies; (2) non-primary gastric origin of tumors; and (3) incomplete clinical data.

For the external validation cohort, patients with missing key predictors or outcome data were excluded. Follow-up data were collected via medical record review and supplemented with telephone interviews when necessary, ensuring completeness of the dataset for validation analyses. After applying the criteria, 74 patients were included in the external validation cohort, with follow-up completed by 31 March 2025 (median follow-up 34 months).

### Outcome and predictor variables

The primary outcome was OS, defined as the time in months from diagnosis to death from any cause. Patients alive at the last follow-up were censored.

Predictor variables included: age (continuous, years, at diagnosis), sex (male or female), chemotherapy (yes or no), lymph node ratio (LNR), T category, M category, tumor size (dichotomized as <5 cm or ≥5 cm according to the study dataset), and tumor number (single [ = 1] or multiple [≥2]).

All patients included in this study underwent radical gastrectomy with lymphadenectomy. To ensure accurate calculation of LNR, only cases with both examined and positive lymph node counts available were retained. Patients with missing nodal information were excluded from the analysis. To avoid collinearity, N category was excluded.

Chemotherapy was defined as perioperative (including neoadjuvant) or adjuvant systemic therapy, both categorized as “yes” due to limitations of the SEER database, which does not reliably distinguish between treatment intent or regimen details. Patients with no recorded chemotherapy were coded as “no.”

Although the definitions of T category and M category have been slightly modified across AJCC editions, their underlying biological meanings—tumor invasion depth and metastatic burden—remain conceptually consistent. Therefore, T and M categories were modeled separately rather than using overall AJCC stage, to enhance comparability and generalizability across cohorts.

### Statistical analyses

Baseline characteristics were summarized using standardized mean differences (SMDs) and chi-square (χ^2^) tests. Age and LNR were treated as continuous variables to avoid bias from data-driven cut points. LNR was modeled using restricted cubic splines (RCS) with four knots to capture potential nonlinearity, while age was retained as a linear term. Other variables (sex, chemotherapy, T category, M category, tumor size, tumor number) were entered as categorical factors.

All predictor variables were initially included in a cox proportional hazard model. Variables lacking independent prognostic value were excluded to obtain a reduced final model.

A nomogram was derived from the reduced final model to estimate individualized 1-, 3-, and 5-year OS. Internal validation used 1,000 bootstrap resamples to correct for optimism and assess discrimination and calibration. External validation focused primarily on 36-month OS, with time-dependent discrimination and calibration evaluated at this horizon. Additional exploratory analyses were conducted at 12 and 60 months to assess model performance over shorter and longer follow-up periods. Inverse probability of censoring weighting (IPCW) was applied to account for censoring in all time-dependent performance metrics.

Model performance was assessed via time-dependent Harrell’s C-index, time-dependent receiver operating characteristic (ROC) curves with area under the curve (AUC; >0.70 considered meaningful), calibration plots (observed vs. predicted survival), and decision curve analysis (DCA) to evaluate net clinical benefit across plausible threshold probabilities. Risk stratification into low, intermediate, and high groups was based on tertiles of nomogram points.

All statistical analyses were conducted using R software (version 4.4.2), and two-sided p < 0.05 was defined statistical significance.

## Results

### Study cohorts

From the SEER database, a total of 2,203 GSRC cases were randomly split into a modeling cohort (70%, n = 1,542) and an internal validation cohort (30%, n = 661). An external cohort of 74 patients from the Second Affiliated Hospital of Nanjing Medical University was used for validation. Baseline characteristics were comparable across cohorts (Table 1).

**TABLE 1:** Baseline characteristics.

Characteristic	Trainingn = 1,542^a^	Internaln = 661^a^	Externaln = 74^a^	p^b^	SMD (Train vs. Internal)^c^	SMD (Train vs. External)^c^
Age (years)	60 (50, 70)	61 (50, 71)	64 (55, 70)	0.440	0.026	0.138
Lymph node ratio	0.12 (0.00, 0.50)	0.13 (0.00, 0.52)	0.13 (0.00, 0.40)	0.793	0.038	0.100
Sex				0.746	0.036	0.004
Female	747 (48%)	332 (50%)	36 (49%)			
Male	795 (52%)	329 (50%)	38 (51%)			
Tumor number				0.574	0.002	0.146
0	1,427 (93%)	612 (93%)	71 (96%)			
≥1	115 (7%)	49 (7%)	3 (4%)			
Tumor size (grouped)				0.482	0.025	0.122
<5 cm	887 (58%)	372 (56%)	47 (64%)			
≥5 cm	655 (42%)	289 (44%)	27 (36%)			
Chemotherapy				0.884	0.004	0.059
No	522 (34%)	225 (34%)	23 (31%)			
Yes	1,020 (66%)	436 (66%)	51 (69%)			
T category				0.255	0.044	0.292
T1	320 (21%)	133 (20%)	24 (32%)			
T2	164 (11%)	72 (11%)	6 (8%)			
T3	498 (32%)	226 (34%)	24 (32%)			
T4	560 (36%)	230 (35%)	20 (27%)			
M category				0.548	0.040	0.065
M0	1,386 (90%)	602 (91%)	65 (88%)			
M1	156 (10%)	59 (9%)	9 (12%)			

^a^ Median (Q1, Q3); n (%).

^b^ Kruskal–Wallis rank sum test; Pearson’s Chi-squared test with simulated p (based on 100,000 replicates).

^c^ SMD: numeric = |Δmean|/SDpooled; categorical = multinomial SMD (Austin 2019).

### Model development and functional forms

**FIGURE 1:**
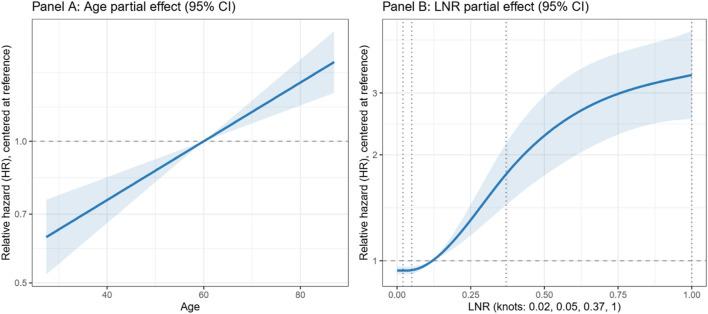
Restricted cubic spline (RCS) partial-effect plots in the modeling cohort. Panel **(A)** Age; Panel **(B)** lymph node ratio (LNR). Curves show relative hazard (log scale) centered at the cohort median of each predictor, with 95% confidence bands. Vertical dotted lines indicate RCS knot locations (LNR knots: 0.02, 0.05, 0.37, 1.00). All other covariates were set to their most frequent category within the modeling cohort.

In the modeling cohort, the LNR (range 0.00–1.00; 5th, 35th, 65th, 95th percentiles 0.02, 0.05, 0.37, 1.00) was highly right-skewed. We modeled LNR with a 4-knot RCS at those percentiles, placing interior knots where risk changes rapidly and anchoring the extremes; nonlinearity versus a linear term was strong (Wald χ^2^ (2) = 74.77, p < 0.001). Age was entered linearly (Wald χ^2^ (1) = 25.32, p < 0.001). Tumor size, chemotherapy, M category, and T category were treated as categorical predictors. Sex and tumor number showed no independent association (sex: Wald χ^2^ (1) = 0.01, p = 0.94; tumor number: Wald χ^2^ (1) = 2.27, p = 0.13); removing them did not worsen fit (LRT χ^2^ (2) = 2.44, p = 0.30; ΔAIC = 1.56) or discrimination (optimism-corrected ΔC≈0.00), and remaining coefficients changed by ≤ 6.60%, so they were excluded. Because extreme LNR values (approaching 1.00) are clinically meaningful, no truncation or winsorization was applied. Partial effect (spline) plots with 95% confidence bands (Figure 1) and Wald statistics (Table 2) document these functional form choices.

**TABLE 2:** LNR was modeled with restricted cubic splines; the row reports the nonlinearity (spline) component.

Term	df	Wald χ^2^	p
Age	1	25.32	<0.001
LNR	2	74.77	<0.001
Sex	1	0.01	0.94
Tumor number	1	2.27	0.13
Tumor size	1	13.59	<0.001
Chemotherapy	1	17.70	<0.001
M category	1	32.39	<0.001
T category	3	108.56	<0.001

### Nomogram derivation and visualization

**FIGURE 2:**
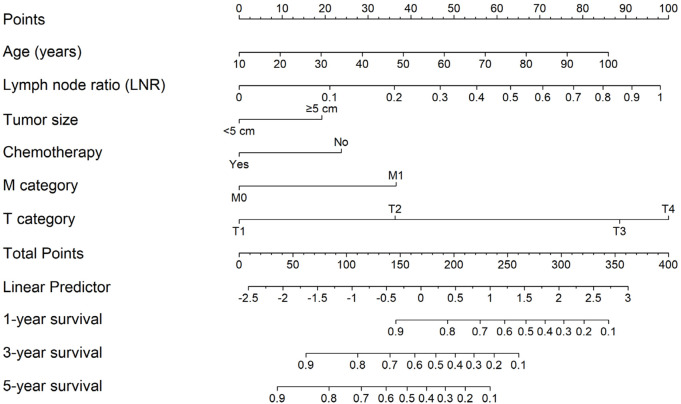
Nomogram for predicting 1, 3, and 5-year survival based on the reduced cox model (age linear; LNR modeled with RCS; tumor size, chemotherapy, M category, and T category as categorical predictors). Points for each predictor are summed to a total score that maps to predicted survival probabilities.

A nomogram was constructed from the reduced cox model (excluding sex and tumor number), in which age entered linearly and LNR was modeled using RCS; tumor size, chemotherapy, T category, and M category were modeled as categorical variables. For each predictor variable, the corresponding point value is read on the top “Points” scale and summed to a “Total Points” score, which maps to the predicted 1-, 3-, and 5-year survival probabilities on the bottom scales (Figure 2).

### Risk stratification

**FIGURE 3:**
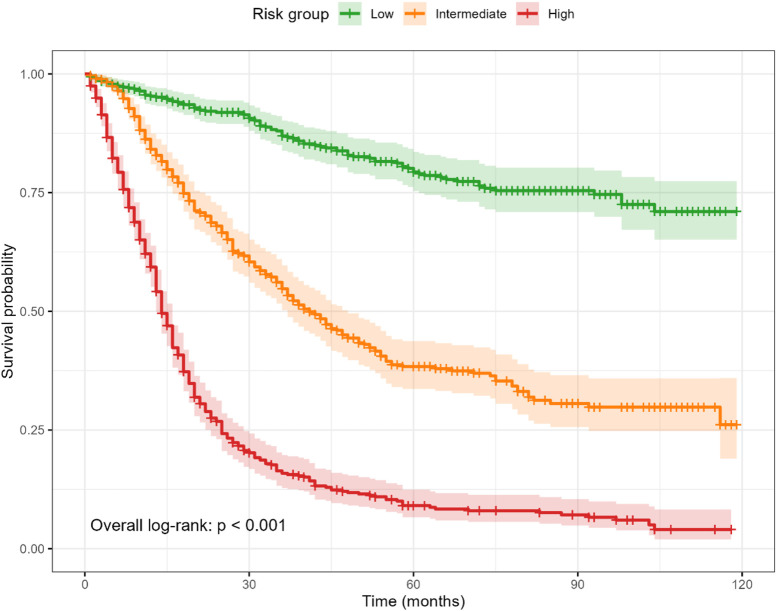
Kaplan–Meier survival by risk tertiles derived from the nomogram total points. Curves show clear, monotonic separation (overall log-rank p < 0.001). Shaded ribbons indicate 95% confidence intervals; time axis is in months.

Patients were stratified into low-, intermediate-, and high-risk groups based on tertiles of the nomogram total points. Kaplan–Meier curves demonstrated clear, monotonic separation (log-rank p < 0.001; Figure 3). Median survival was not reached in the low-risk group, while it was 41 months in the intermediate-risk group and 14 months in the high-risk group. Compared with the low-risk group, the hazard ratios for mortality were 3.93 (95% CI, 3.11–4.98) in the intermediate-risk group and 11.50 (95% CI, 9.16–14.45) in the high-risk group.

### Clinical utility (DCA)

**FIGURE 4:**
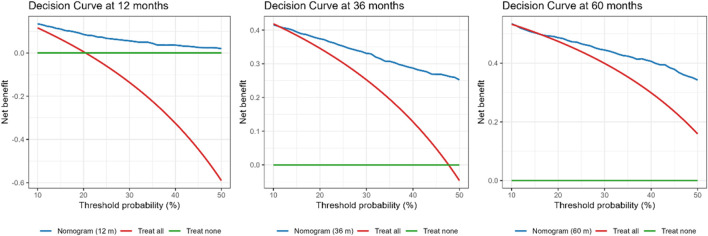
DCA comparing the nomogram with “treat all” and “treat none” strategies at 12, 36, and 60 months. The nomogram provided higher net benefit across thresholds of 10%–50% at 12 months (peak gain 8.2% at 21%), 12%–50% at 36 months (peak gain 26.2% at 48%), and 10%–11% and 17%–50% at 60 months (peak gain 18.4% at 50%). Net benefit is standardized; threshold probability is the risk cut-off at which an intervention would be offered.

DCA showed net benefit for the nomogram than “treat-all” or “treat-none” strategies across clinically relevant thresholds. At 12 months, the nomogram exceeded both strategies for thresholds 10%–50%, with a peak gain of 8.20% at a 21% threshold. At 36 months, net benefit was higher across thresholds 12%–50%, peaking at 26.20% at 48%. At 60 months, the nomogram outperformed both strategies at thresholds 10%–11% and 17%–50%, with a peak gain of 18.40% at 50% (Figure 4), supporting its clinical utility over a broad range of decision thresholds.

### Internal validation

**FIGURE 5:**
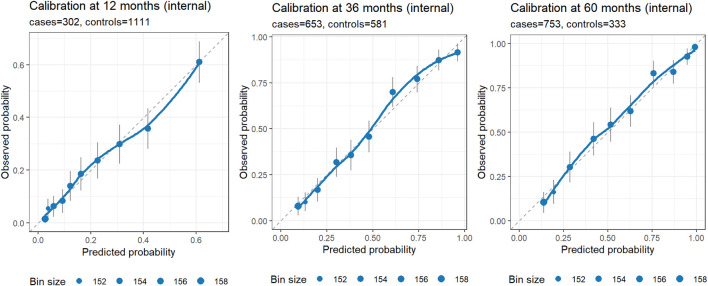
Internal calibration at 12, 36, and 60 months. Each panel displays observed risk (Kaplan–Meier estimate at the horizon) versus predicted risk (1 – S (t | lp) from the rms model) within quantile-based bins of predicted risk (default 10 bins). Points mark bin averages, with point size proportional to bin size; vertical error bars denote approximate 95% CIs; the dashed diagonal indicates perfect calibration; and the LOESS curve summarizes local calibration trends. Axes are on the same 0–1 scale to facilitate visual comparison. Panel subtitles report the number of cases and controls at each horizon. Closer alignment of points/LOESS to the diagonal indicates better calibration; wider CIs or deviations at probability extremes should be interpreted in light of the available events/controls in those regions.

Beyond clinical utility demonstrated by DCA, the nomogram showed strong discrimination. Harrell’s C-index was 0.78 (95% CI 0.76–0.79) on apparent data and 0.77 after bootstrap correction (optimism = 0.00). Time-dependent AUCs at 12, 36, and 60 months were 0.79 (95% CI 0.76–0.82), 0.85 (95% CI 0.83–0.88), and 0.85 (95% CI 0.83–0.88), respectively. Calibration at 1, 3, and 5 years showed close agreement between predicted and observed probabilities after bootstrap correction (Figure 5), indicating reliable internal performance.

### External validation

**FIGURE 6:**
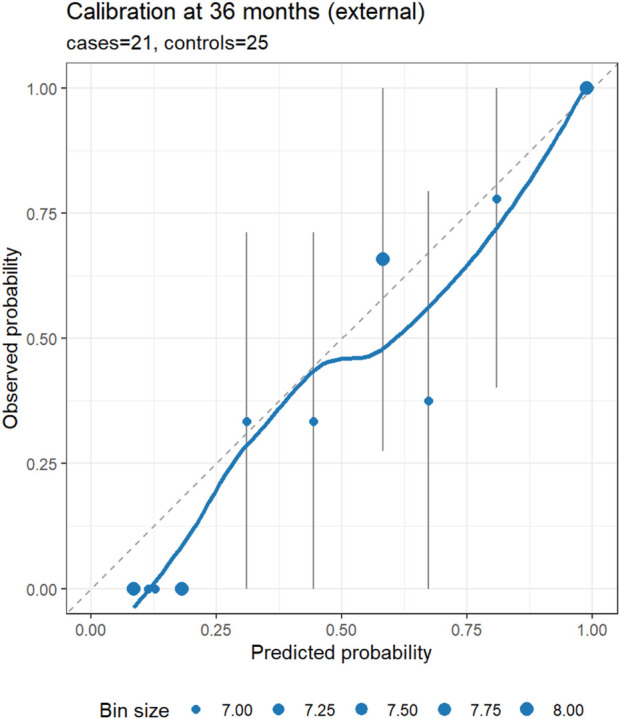
External calibration of the nomogram at 36 months (primary horizon). Points show observed risk (Kaplan–Meier estimate at 36 months) versus predicted risk (1 – S (t | lp)) within bins of predicted risk; vertical bars denote approximate 95% CIs; the dashed line indicates perfect calibration; and the LOESS curve summarizes local trends. The panel subtitle reports the number of cases and controls at the horizon.

**FIGURE 7:**
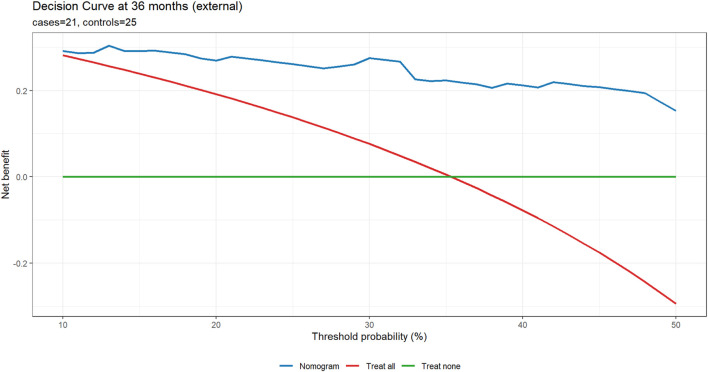
External DCA at 36 months (primary horizon). Net benefit is plotted against the threshold probability (10%–50%) for three strategies: Nomogram, Treat all, and Treat none, using time-to-event DCA at the specified horizon. Panel subtitles report the number of cases and controls.

In the external cohort, we specified 36 months as the primary horizon and treated 12 and 60 months as exploratory, using IPCW for time-dependent metrics. At 36 months (21 cases/25 controls), the nomogram showed strong discrimination (IPCW-AUC 0.91) and lower prediction error (IPCW-Brier 0.13 vs. null model 0.23; absolute reduction 0.10, relative improvement 43.5%). Uno’s C-index was 0.79. Recalibration indicated under-dispersion of the linear predictor: the cox recalibration slope was 2.02 (95% CI 1.28–2.75) with a log (−log) intercept of −1.63 (ideal: slope 1.00, intercept 0.00). Logistic recalibration of 36-month risk yielded a slope of 1.62 (95% CI 0.81–2.85) and an intercept of −0.28 (95% CI −1.17–0.58), suggesting no major systematic shift in average absolute risk despite slope >1. The integrated Brier score over 0–36 months (IBS) was 0.07, indicating acceptable average prediction error across the interval. At exploratory horizons (12 and 60 months), IPCW-AUCs were 1.00 (1.00–1.00) at both time points; these apparent “perfect” values reflect extreme class imbalance (12 months: 6/64; 60 months: 24/6) rather than genuinely flawless discrimination and warrant cautious interpretation. Corresponding Brier scores were 0.19 vs. 0.08 (null) at 12 months and 0.06 vs. 0.25 (null) at 60 months. Calibration and DCA at 36 months are shown in Figures 6, 7.

## Discussion

While GSRC is considered to be poorly differentiated compared to other histological types of GC, the prognosis of GSRC is still debated and appears to depend on the stage of the cancer at the time of diagnosis (Zhang S. et al., 2021). For early GC, defined by the Japanese Gastric Cancer Association (JGCA) as gastric carcinoma confined to the mucosa or submucosa, regardless of the presence or absence of lymph node metastasis, the prognosis of GSRC has been reported in various studies to be comparable to, or even better than, that of other types of gastric adenocarcinoma (Japanese Gastric Cancer Association, 2011). Conversely, in advanced GC, the prognosis of GSRC is more controversial and is commonly thought to be poor (Kim et al., 2004; Ha et al., 2008; Pernot et al., 2015). However, Zhao et al. (2021) reported that the OS of GSRC patients was insignificantly different from that of non-GSRC patients. Therefore, this study aims to identify prognostic factors of GSRC patients and to develop a nomogram based on these factors, in order to support early prevention and prognosis evaluation for GSRC patients.

This study included age, LNR, T category, M category, chemotherapy, tumor size as predictor variables. These factors are consistent with previously reported prognostic factors for GC and especially GSRC (Chen YF. et al., 2023; Chen YR. et al., 2023). Among them, LNR emerged as a particularly strong predictor of survival, which is defined as the ratio of metastatic lymph nodes (LN) over total LN examined. Previous study has similarly pointed out LNR as a superior metric compared to the traditional N stage alone (Zhang M. et al., 2021). Lee et al. (2001) pointed out that the N stage is affected by the number of lymph nodes removed (RLNs), which can cause stage migration if RLNs are insufficient. Zhang et al. (2023) demonstrated that LNR is superior to the N stage regardless of early or advanced GSRC, and is an independent risk factor associated with patient outcomes. The NCCN guidelines indicate that the removal of an adequate number of lymph nodes (≥15) is not only beneficial for staging but also positively influences the survival in patients with advanced (Ajani et al., 2025). Insufficient lymph nodes retrieval—particularly when fewer than 15 nodes are examined—can lead to the prognosis of GC patients being underestimated. These results indicated that a sufficient number of lymph node biopsies are required and beneficial to precisely calculate LNR, which in turn stages the tumor and guides appropriate postoperative management (Zhao et al., 2016).

Several limitations must be acknowledged. We required both examined and positive lymph node counts to compute LNR, and excluded records with incomplete nodal information. While this enhances internal consistency and enables LNR-based modeling, it may introduce selection bias by overrepresenting patients who underwent more extensive or better-documented lymphadenectomy. Such patients may differ systematically from those with limited nodal assessment, potentially inflating apparent performance. We depict the patient flow in Supplementary Figure S1.

The external cohort was small (n = 74) with limited follow-up (median 34 months), leading to sparse event counts at early and late horizons and unstable time-dependent metrics. The extreme class imbalance explains the apparent “perfect” IPCW-AUCs at 12 and 60 months; these should not be overinterpreted. We therefore focused the primary external evaluation on 36 months and downgraded 1- and 5-year findings to exploratory.

In addition, SEER database lacks key variables that influence prognosis and treatment selection, including chemotherapy regimens (agents, cycles, dose intensity), surgical details (extent of lymphadenectomy such as D1 vs. D2, margin status beyond R0 coding granularity), patient performance status, comorbidities and molecular markers. This absence substantially limits transportability and practical application in heterogeneous clinical settings where these factors guide decision-making and affect outcomes. The nomogram should be understood as providing a baseline prognostic estimate; for individual patient decisions, its predictions must be integrated with these critical, unmeasured clinical factors by the treating physician. Future models that incorporate molecular features may improve performance and personalization.

Finally, because chemotherapy receipt is likely confounded by indication, we report associations rather than causal effects. Causal inference would require richer treatment details, time-varying confounders, and prospective designs.

## Conclusion

In conclusion, we constructed a nomogram to predict OS of GSRC patients. This model offers an effective tool for survival prediction and can support clinical decision-making in the management of GSRC. Further prospective and multi-center validation is needed to strengthen its utility in routine practice.

## Data Availability

The SEER database is publicly available at the Surveillance, Epidemiology, and End Results (SEER) Program (https://seer.cancer.gov/). Researchers can obtain access by submitting a data request through the SEER*Stat system. The Chinese registry dataset was obtained from The Second Affiliated Hospital of Nanjing Medical University. Due to patient privacy and institutional regulations, it is not publicly available. Access may be granted upon reasonable request and approval from the institutional review board.
