# Supplementary material for: A prognostic nomogram for predicting overall survival in gastric signet ring cell carcinoma patients: a SEER database and Chinese registry analysis
Source: Front Mol Biosci. 2025 Nov 17;12:1704157. doi: 10.3389/fmolb.2025.1704157 (PMC12665595; doi:10.3389/fmolb.2025.1704157)

**Supplementary Methods: Model Specification and Prediction Equation**

To ensure reproducibility and external application, we provide the full multivariable coefficients, baseline survival estimates, and the exact prediction equation of the final Cox proportional hazards model.

**1. Full multivariable coefficients**

Table S1 reports the estimated regression coefficients (β), standard errors, Wald statistics, p-values, 95% confidence intervals, and hazard ratios (HRs) for all covariates. Age was modeled as a linear term (per year; HR ≈ 1.16 per 10-year increase). Lymph node ratio (LNR) was modeled using restricted cubic splines with 4 knots (0.02, 0.05, 0.37, and 1.00); the three spline basis functions (LNR, LNR_rcs2, LNR_rcs3) act jointly and should not be interpreted individually. Tumor size, chemotherapy, M category, and T category were included as categorical variables with reference levels explicitly indicated.

Table S1

| **Term** | **Coefficient (β)** | **SE** | **z** | **p** | **95% CI (β)** | **exp(β)** | **95% CI (exp(β))** | **Note** |
| --- | --- | --- | --- | --- | --- | --- | --- | --- |
| Age (per year) | 0.015 | 0.003 | 5.249 | <0.001 | 0.009 – 0.020 | 1.015 | 1.009 – 1.021 | Linear |
| LNR (spline base) | 3.533 | 1.164 | 3.034 | 0.002 | 1.251 – 5.814 | — | — | RCS component |
| LNR_rcs2 | -64.254 | 54.676 | -1.175 | 0.240 | -171.417 – 42.909 | — | — | RCS component |
| LNR_rcs3 | 69.861 | 60.786 | 1.149 | 0.250 | -49.278 – 189.000 | — | — | RCS component |
| Tumor size ≥5 cm | 0.300 | 0.079 | 3.809 | <0.001 | 0.145 – 0.454 | 1.349 | 1.157 – 1.575 | vs <5 cm |
| Chemotherapy Yes | -0.370 | 0.086 | -4.299 | <0.001 | -0.538 – -0.201 | 0.691 | 0.584 – 0.818 | vs No |
| M1 | 0.569 | 0.101 | 5.619 | <0.001 | 0.370 – 0.767 | 1.766 | 1.448 – 2.154 | vs M0 |
| T2 | 0.565 | 0.207 | 2.725 | 0.006 | 0.159 – 0.972 | 1.760 | 1.172 – 2.643 | vs T1 |
| T3 | 1.378 | 0.168 | 8.188 | <0.001 | 1.049 – 1.708 | 3.969 | 2.853 – 5.520 | vs T1 |
| T4 | 1.556 | 0.170 | 9.150 | <0.001 | 1.223 – 1.889 | 4.741 | 3.397 – 6.616 | vs T1 |

**2. Baseline survival and cumulative hazard**

The baseline survival function $S_{0}\left( t \right)$(corresponding to all covariates at reference levels, Age = 0, and LNR = 0) and the corresponding cumulative hazard $H_{0}(t)=-ln\left[ S_{0}\left( t \right) \right]$ were estimated at 12, 36, and 60 months (Table S2). These estimates allow computation of absolute survival probabilities for new patients using:

$S(t\mid X)=S_{0}\left( t \right)^{exp\left( LP \right)},H(t\mid X)=H_{0}(t)\cdot exp(LP)$,

where LP is the linear predictor defined below.

Table S2

| Time (months) | $S_{0}\left( t \right)$ | $H_{0}\left( t \right)=-ln\left[ S_{0}\left( t \right) \right]$ |
| --- | --- | --- |
| 12 | 0.859263 | 0.151681 |
| 36 | 0.572257 | 0.558168 |
| 60 | 0.429140 | 0.845971 |

**3. Exact prediction equation**

Restricted cubic spline basis functions were constructed as follows (knots: k1=0.02, k2=0.05, k3=0.37, k4=1.00):

$$g\left( x,k \right)=\left( x-k \right)_{+}^{3}, D_{j}\left( x \right)=\frac{g\left( x,k_{j} \right)-g\left( x,k_{4} \right)}{k_{4}-k_{j}},j=1,2,3$$

$$Z_{1}\left( x \right)=x, Z_{2}\left( x \right)=D_{2}\left( x \right)-D_{3}\left( x \right), Z_{3}\left( x \right)=D_{1}\left( x \right)-D_{3}\left( x \right).$$

The linear predictor (LP) is given by:

$$LP=0.01486\boldsymbol{\times}Age\boldsymbol{+}3.5327\boldsymbol{\times}Z_{1}(LNR)\boldsymbol{-}64.2537\boldsymbol{\times}Z_{2}(LNR)\boldsymbol{+}69.8607\boldsymbol{\times}Z_{3}(LNR)\boldsymbol{+}0.2997\boldsymbol{\times}I(Tumor size\geq5cm)-0.3697\times I(Chemotherapy = Yes)+0.5689\times I(M1)+0.5653\times I(T2)+1.3785\times I(T3)+1.5562\times I(T4).$$

Figure S1


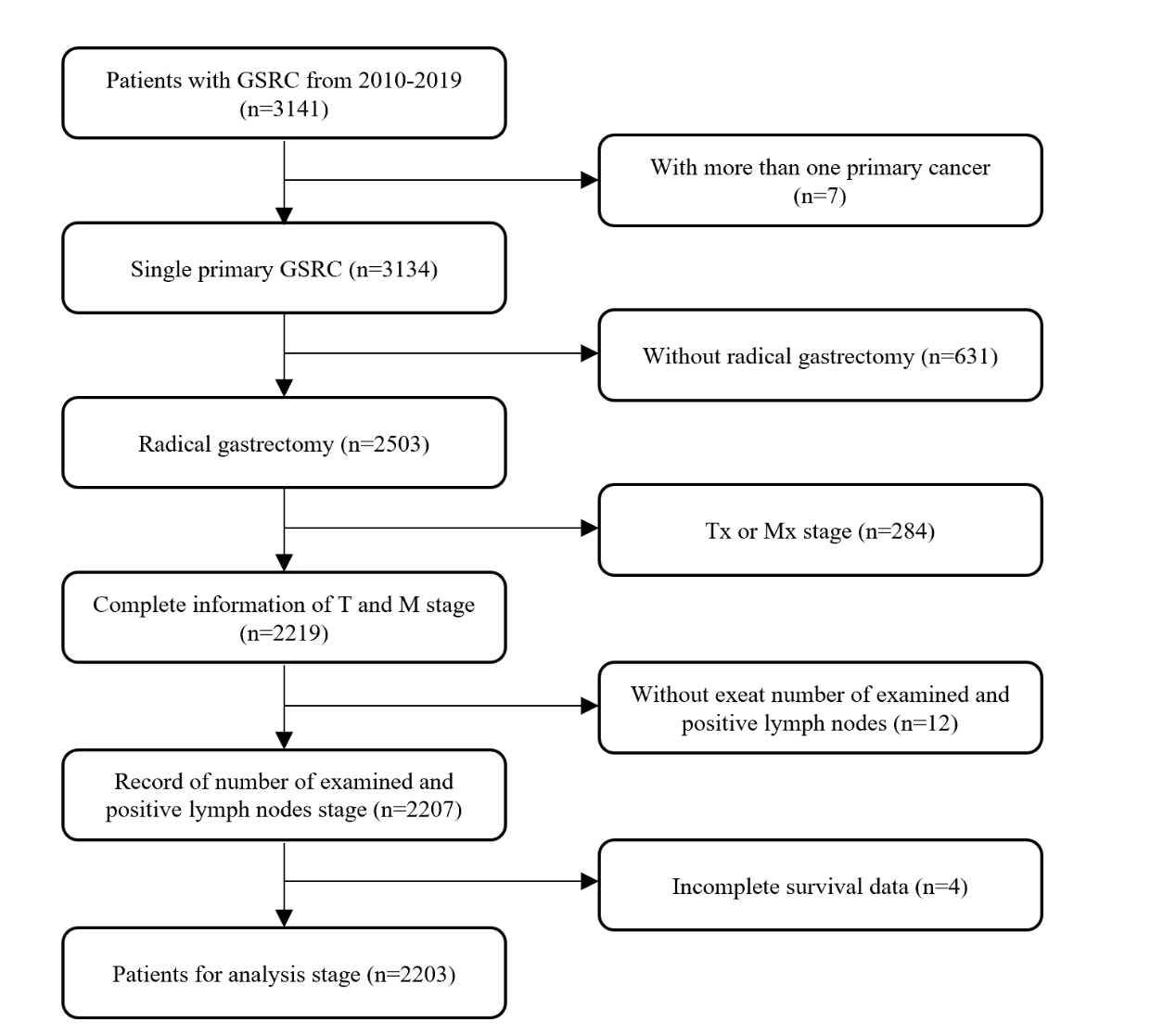

Supplement: Supplementary file 1 [file Supplementaryfile1.docx]
